# Is Tobacco an Antiseptic?

**Published:** 1882-08

**Authors:** Frank French

**Affiliations:** Rochester, N. Y.


### ARTICLE. IV.

### *Is Tobacco an Antiseptic?*

BY FRANK FRENCH, D. D. S., ROCHESTER, N. Y.

[Read before the Seventh District Dental Society April, 1882]

"When all things were made, none was made better than tobacco, to be a lone man's companion, a bachelor's friend, a hungry man's food, a sad man's cordial, a wakeful man's sleep, and a chilly man's fire; while for stanching of wounds, purging of rheum, and settling of the stomach, there's no herbe like it under the canopy of heaven." So spake old Salvation Yeo many long years ago, and thousands to-day will say amen ! to it.

Tobacco, that "precious stinke," as his vindictive majesty King James, called it, first became known to Europeans shortly after the discovery of America. All its present popular uses were probably known to the natives of North and South America, ages before Columbus was born. When the Spaniards landed in Paraguay, in 1503, the natives came forth to oppose them, "beating drums, throwing water, and chewing tobacco and spirting the juice from their mouths upon the invaders" which as a means of defense and offense, must have been a painful surprise to the Spaniards, if the Indians had anything of the accuracy of aim said to be attained by some of our western friends.

Tobacco was no sooner introduced, than it was seized upon by the medical faculty as a valuable addition to their pharmacopia. In a volume published by Henry Butler he says "it cureth any griefe, dolour, imposthume, or obstruction, proceeding of colde or winde, especially in the head or breast. The fume taken in a pipe is good against rumes, catarrhs, hoarseness, ache in the head, stomach, lungs, breast; also in want of meate, drinke, sleepe or reste." But very soon its opponents began a war against it and the battle which began two centuries ago continue still. King James imposed the first tax on tobacco: in Russia smoking was punished by amputation of the nose; in the Swiss Canton of Berne the offense ranked next to adultery. Innocent XII in 1690 solemnly excommunicated all who should take snuff or tobacco in church.

It is not my purpose to extol tobacco; and I love it too well to decry it, so that what I have to say will be in its connection with our specialty, and is by no means intended as a tirade against it. I have tried to destroy as much of it as possible by burning it and I expect to keep trying as long as I live.

Tobacco contains nicotine in certain proportions, which is of itself *after* distillation, alkaline. As it exists in tobacco it is combined in *excess* with acid—10,000 parts of fresh leaves containing only six parts of nicotine, but fifty-one parts of mallic acid, and nine parts of nitrid acid combined with other substances. In its concentrated form its action upon the animal system is one of the most virulent poisons known, one drop being sufficient to kill a dog, and destroying life in man in poisonous doses in from two to five minutes. Havana tobacco contains 2 per cent. of nicotine, Maryland 2.3 per cent,, and Virginia 6.9 per cent., but these are from fresh leaves, and tobacco undergoes considerable chemical change in the process of curing, it being asserted that there is a much greater proportion of nicotine in prepared tobacco, than in fresh leaves. A fermentation takes place which converts certain pre-existing principles into nicotine. Another change is produced by burning it, as high as 9 per cent. of nicotine having been obtained by this process. The odorous principle of tobacco is said to be due to nicotine. The smoke contains Sulphuretted Hydrogen and Hydrocianic Acid in small quantities.

In connection with many others, when asked by my patients in regard to the effects of tobacco upon the teeth, I have been accustomed to say it was not injurious, where ordinary cleanliness was observed ; that it was a well known fact that those who used tobacco suffered less with their teeth than those who did use it, that it was antiseptic in its nature. I still think it is antiseptic, but I think I have proven by experiments during the past few weeks, that it is not antacid. My attention was called to it about a year ago while operating for a physician. He asked me what effect tobacco had on the teeth and I replied that it was antiseptic. He called my attention to the first right inferior molar and bicuspids, each of which was decayed on the labial surface at the neck of the tooth. I told him it was caused by chemical action from acid formations. He asked why on these and on none of the other teeth, and told me that he felt sure it was caused by tobacco, as that was where he always carried it, and that he should discontinue the use of it. He was about 40 years old, had splendid teeth, took excellent care of them, and none of the others showed the least symptom of decay. About six months ago a gentleman, sixty-three years old, came in to have his teeth cleaned. He had the full number of teeth, 32, all of them perfectly sound except first right inferior molar and bicuspids, which were decayed at the margin of the gums, the same as the other case mentioned. I called his attention to them and he at once said, "Oh, that is where I used to carry my tobacco. I used it for forty years but have quit now."

Another case of a young man who had only chewed tobacco a year, and the molar and bicuspids were affected the same as the other cases mentioned. All three of these persons chewed what is called the "best fine cut."

I then began trying experiments, and resolved to call the attention of the society to it, that others might investigate, and also to give the result of my experiments. My plan has been to place a portion of the article to be tested in a small vial with pure water, cork it, let it remain over night and then test it for acid. It might be said that this is hardly as fair a test as it would have been to place it in saliva, but it must be remembered that saliva contains a small percentage of acid, and that in testing it you are far more apt to get an acid reaction than an alkaline, or even neutral. So that using pure water for the infusion, you probably get *less* acid reaction than if saliva were used. I have tried a great many different brands of cigars and in nearly every case there has been an acid reaction, some veay marked and others only a trace; the cheap cigars showing far more acid than the higher priced ones. None of the cheap cigars but that showed acid distinctly, (when I say cheap cigars I mean five centers) while in many of the higher priced ones there was only a trace, and a very few, none at all. The moral of this is, do not buy poor cigars. Smoking tobacco I have not tested at all, as this does not come in contact with the teeth. Chewing tobacco gave by far the strongest acid reaction; being marked in every case, and the very best was as strong as the poorest. One sample of what was said to be the very best in the market, after being placed over night in a vial with water, on touching the cork in the motning, it flew out with a pop, and effervesced like a bottle of beer. It turned litmus paper a bright scarlet at once. There, gentlemen, are the results of my experiments. I may add that I have smoked for 30 years and the upper tooth where I always hold my cigar, lost its vitality five or six years ago, but the lower one is perfectly sound, A friend who is an inveterate smoker, has lost entirely by gradual crumbling, the upper tooth where he held his cigar, while the lower one is all right. I do not say that tobacco had anything to do with either of us in producing this result. What think ye?
—*Odontographic Journal.*

---

